# Bioactivities from Marine Algae of the Genus *Gracilaria*

**DOI:** 10.3390/ijms12074550

**Published:** 2011-07-15

**Authors:** Cynthia Layse F. de Almeida, Heloina de S. Falcão, Gedson R. de M. Lima, Camila de A. Montenegro, Narlize S. Lira, Petrônio F. de Athayde-Filho, Luis C. Rodrigues, Maria de Fátima V. de Souza, José M. Barbosa-Filho, Leônia M. Batista

**Affiliations:** Department of Pharmaceutical Sciences, Laboratory of Pharmaceutical Technology, Federal University of Paraiba, João Pessoa, PB 58051-900, Brazil; E-Mails: cynthialayse@gmail.com (C.L.F.A.); heloinafalcao@yahoo.com.br (H.S.F.); gedson@ltf.ufpb.br (G.R.M.L.); camila_montenegro@ltf.ufpb.br (C.A.M.); narlizelira@yahoo.com.br (N.S.L.); athayde-filho@quimica.ufpb.br (P.F.A.-F); lcezar@ltf.ufpb.br (L.C.R.); mfvanderlei@ltf.ufpb.br (M.F.V.S.); jbarbosa@ltf.ufpb.br (J.M.B.-F.)

**Keywords:** *Gracilaria*, macroalgae, seaweed, biological activity, natural product, review

## Abstract

Seaweeds are an important source of bioactive metabolites for the pharmaceutical industry in drug development. Many of these compounds are used to treat diseases like cancer, acquired immune-deficiency syndrome (AIDS), inflammation, pain, arthritis, as well as viral, bacterial, and fungal infections. This paper offers a survey of the literature for *Gracilaria* algae extracts with biological activity, and identifies avenues for future research. Nineteen species of this genus that were tested for antibacterial, antiviral, antifungal, antihypertensive, cytotoxic, spermicidal, embriotoxic, and anti-inflammatory activities are cited from the 121 references consulted.

## 1. Introduction

The ocean environment contains over 80% of world’s plant and animal species [1] and with more than 150,000 seaweeds found in the intertidal zones and tropical waters of the oceans, it is a primary source of natural products [2].

Seaweeds are floating and submerged plants of shallow marine meadows. They have salt tolerance because the osmolarity of cytoplasm is adjusted to match the osmolarity of the seawater so that desiccation does not occur. They lack true stems, roots and leaves; however, they possess a blade that is leaf like, a stipe that is stem like, and a holdfast that resembles roots like terrestrial plants. Seaweeds contain photosynthetic pigments and use sunlight to produce food and oxygen from carbon dioxide, and the water [3].

Marine macroalgae are important ecologically and commercially to many regions of the world, especially in Asian countries such as China, Japan and Korea [4]. They are a valuable food resource which contains low calories, and they are rich in vitamins, minerals, proteins, polysaccharides, steroids and dietary fibers [5–7]. Since as early as 3000 BC, they were also considered important as traditional remedies [4]. The Japanese and Chinese use brown algae in the treatment of hyperthyroidism and other glandular disorders [8–11]. The unsaturated lipids afford protection against cardiovascular pathogens [12].

Seaweeds have been one of the richest and most promising sources of bioactive primary and secondary metabolites [13] and their discovery has significantly expanded in the past three decades [4,14,15]. The algae synthetize a variety of compounds such as carotenoids, terpenoids, xanthophylls, chlorophyll, vitamins, saturated and polyunsaturated fatty acids, amino acids, acetogenins, antioxidants such as polyphenols, alkaloids, halogenated compounds and polysaccharides such as agar, carrageenan, proteoglycans, alginate, laminaran, rhamnan sulfate, galactosyl glycerol and fucoidan [16–25].

These compounds probably have diverse simultaneous functions for the seaweeds and can act as allelopathic, antimicrobial, antifouling, and herbivore deterrents, or as ultraviolet-screening agents [26]. They are also used by the pharmaceutical industry in drug development to treat diseases like cancer, acquired immune-deficiency syndrome (AIDS), inflammation, pain, arthritis, infection for virus, bacteria and fungus [27]. Currently, algae represent about 9% of biomedical compounds obtained from the sea [28].

Compounds with cytostatic, antiviral, antihelmintic, antifungal and antibacterial activities have been detected in green, brown and red algae [29,30]. The algae produce pure forms of the fatty acids found in human milk that appear to be building blocks for mental and visual development [31] and have been extensively screened for syntheses of new drugs [32,33].

During the 1970s, Ryther and collaborators evaluated numerous species of red, green and brown macroalgae for their potential growth rates and dry weight yields [34]. They demonstrated that the genus *Gracilaria* was the most attractive candidate because of its ability to achieve high yields and while producing commercially valuable extracts [35].

*Gracilaria* Greville genus (Gracilariales, Rhodophyta) is a macroalgae group with more than 300 species of which 160 have been accepted taxonomically. These are usually red, green or greenish brown with a three-phase cycle and can be found in tropical and subtropical seas [36,37].

The *Gracilaria* species are important for the industrial and biotechnological uses because they have phycocolloids, the main source of agar α-(1,4)-3,6-anhydro-l-galactose and β-(1,3)-d-galactose with little esterification in cell wall [2,38]. Among the carbohydrates, agar and other polysaccharides are present in *G. confervoides* [39], *G. dura* [40], *G. chilensi* and *G. secundata* [41,42].

These algae also produce important bioactive metabolites like the primary compound with antibiotic activity acrylic acid [43], and the eicosanoids which are derivatives C_20_ polyunsaturated fatty acid (PUFA) metabolism through oxidative pathways that originate mainly from arachidonic acid and eicosapentaenoic acids, the precursors of prostaglandins (PGs) [44,45]. Species such as *G. asiatica* and *G. lichenoids* contain PGE_2_ [46,47]. PGF_2_ and 15-keto-PGE_2_ were respectively isolated from *G. lichenoids* and *G. asiatica* [45]; *G. verrucosa* contains PGA_2_ that appears to be responsible for a gastrointestinal disorder, known as “ogonori” poisoning in Japan [48].

Lipids are abundant in this genus being mainly prostaglandins [49], steroids, such as cholesterol and clinoasterol are present in *G. crassa* and *G. coronopifolia* respectively [50–52], as well as *G. longa* [48,53–57] and *G. dura*. Other steroids such as 3-beta-hydroxy-poriferast-5-en-7-one, 3-beta-7-alpha-diol-poriferast-5-ene and 5-alpha-poriferast-9(11)-en-3-beta-ol are isolated from *G. dura* [50]; cholestane-3-β-5-diol,5-α:24(S)-ethyl [52], poriferastene 8 [50], poriferast-5-ene-3-β-7-β-diol [51] and poriferast-5-ene-3-β-7-α-diol [51] were identified in *G. coronopifolia; G. longa* also has a variety of compounds like alpha linolenic acid, gamma linolenic acid [58], glycolipids [59], 5-dehydro avenasterol, fucosterol, myristic acid, desmosterol and 5-alpha-24(S)-ethyl-cholestane-3-beta-6-beta-diol [60]. Phytochemical studies with extracts from fresh thallus of *G. andersoniana* showed the following isolates: oleic acid, linoleic acid, cholesterol, prostaglandin A_2_, prostaglandin E_2_, leukotriene B_4_ and phytol [61–63].

Studies with *G. asiatica* reported the diterpenes cis and trans-phytol [63]. A variety of lactones are present in *Gracilaria* from the Pacific Ocean, such as aplysiatoxin isolated from *G. confervoides* [64,65], polycavernoside B, polycavernoside B_2_, and polycavernoside A_2_ and A_3_ isolated from *G. crassa* [49,66]. Other constituents are also containedin this genus such as proteins r-phycoerythrin from *G. salicornia* [67] and *G. longa* [68], gigartinine from *G. chilensis* [69] and proteoglycan from *G. longa* [70].

The possibility of finding new molecules from natural products is immeasurable. For this reason the plants and their derivatives are major sources of all drugs, affecting about 30% of pharmaceutical market [71]. According to Newman *et al.* (2003), between the years 1981 and 2002, 877 new molecules were introduced into the market, with 49% of substances isolated from natural sources followed by semi-synthetic derivatives or synthesized molecules taking the structures of natural origin as models [29].

The search for new effective medicines remains a challenge for scientists. Therefore around the world, many researchers have focused on natural sources for new molecules with algae among the targets of these studies. So in this study we reviewed the literature related to bioactivities for *Gracilaria* algae.

## 2. Results and Discussion

In this review, among the 160 species of *Gracilaria* already identified taxonomically, only 19 of them had their extracts and fractions chemically tested for toxicity, cytotoxic, spermicidal, antiimplantation, antibacterial, antiviral, antifungal, antiprotozoa, antihypertensive, antioxidant, anti-inflammatory, analgesic, and spasmolytic effects in gastrointestinal tract (Table 1).

These biological studies were mainly developed in Japan and Brazil. This fact is justified by the extensive coastlines and marine biodiversity and is influenced by several factors for the development of these species, such as temperature, radiation, salinity, metal ions and other chemically fundamental components. Australia and Guam have recently become interested in the study of algae and diverse marine species. The consumption of algae has increased in European countries in recent decades with 15 to 20 species of algae being marketed in Italy, France and Greece. In western countries like Venezuela, USA and Canada, the macroalgae are industrially used as a source of hydrocolloids agar, carrageenan and alginate [100]. Carrageenan has been found to be useful in ulcer therapy and alginates are known to prolong the period of activity of certain drugs [8–11].

### 2.1. Studies of Toxicity

**Figure 1:**
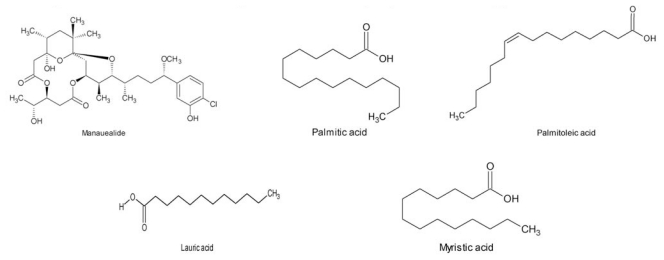
Structure of the compounds found in *G. foliifera*, *G. coronopifolia* and *G. edulis*.

In France, extract studies with ethanol/water draw up from dried entire plant of *G. foliifera* showed toxicity in humans when treated with oral dose and cytotoxicity studies [75,76]. *G. coronopifolia* and *G. edulis* were also toxic to humans [65,49] (See Table 1). Carbohydrate, heparin [97], agar [101], manauealide A, manauealide B [64], manauealide C [102], palmitic, palmitoleic, oleic, lauric and myristic acids [103], steroids and alkaloids malyngamide [104] were found in these species (Figure 1).

There is currently a tendency to substitute the use of laboratory animals in toxicological tests with alternative methods to reduce their numbers in experiments, or refine the existing methodology in order to minimize pain and stress [105]. A rapid and effective alternative to realize primary toxicity and biological action screening of compounds is the estimation of the 50% lethal concentration (LC_50_) through brine shrimp assay using *Artemia salina* L. [106]. A 90% ethanol extract of *G. domingensis* had LC_50_ of 200 μg/mL against *A. salina* [74].

**Figure 2:**
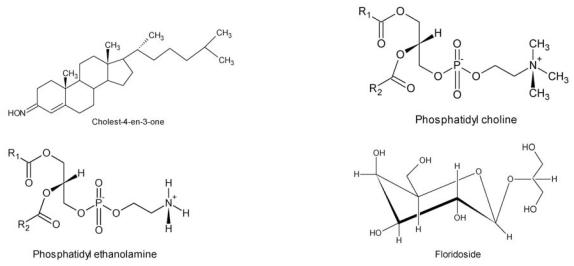
Structure composed of species of *Gracilaria* tested in cytotoxicity.

Another method to evaluate toxicity is determining cytotoxic activity. In this context aqueous extract from dried thallus of *G. bursa-pastoris* (10.0 μg/mL), chloroform and methanol extracts from *G. textorii,* which was isolated steroid cholest-4-en-3-one [107], and ethanol extract from *G. verrucosa* were not toxic in cell culture. However, aqueous extract from *G. verrucosa* at a dose of 1.2 mg/animal showed toxicity to mice [48], according to Table 1. In this seaweed, lipids were indentified, such as PGF_α_ [84,85], glycerol, ethanolamine-phosphatidyl [58], choline-phosphatidyl [58,108], ethanol-phosphatidyl [58], floridoside [109], and carbohydrates, such as agar [110–113] (Figure 2).

### 2.2. Effects on the Nervous System

Studies related to nervous system are important to understanding and treat complex degenerative and behavioral diseases. 90% ethanol extracts from *G. corticata, G. edulis* and *G. verrucosa* did not cause central or periphery effects for mice or dogs (50 mg/kg), and did not show analgesic or anticonvulsant activities for mice [75] (Table 1).

### 2.3. Contraception Activity

The researchers have also investigated new molecules with anticonceptive action; the post-coital contraceptive action of marine seaweeds was also evaluated in animals. Methanol: methylene chloride (1:1) extract from *G. corticata* was orally administered at 500 or 1000 mg/kg/day to female rats from day 1 to day 7 of their pregnancies. Higher doses produced significant post-coital contraceptive activity due to enhanced pre-implantation without any marked side effects. These findings indicate that red marine algae are a potential source for post-coital contraceptive drugs [80].

90% Ethanol extracts from *G. edulis* (100 mg/kg) and *G. corticata* were inactivated before the antiimplantation effect when they tested in pregnant rats [75,80]. Ethanol extracts from shade dried thallus of *G. edulis* and *G. verrucosa* were inactive in spermicidal bioassays [75]. Extracts from *G. edulis* showed 100% inhibition of sperm motility and this effect was related to disruption of the plasma membrane by spermicidal compounds [3] (Table 1).

### 2.4. Anti-Inflammatory and Antioxidant Activities

The anti-inflammatory activity of seaweeds has been studied. Polysaccharide fractions from *G. verrucosa* at a dose of 4.0 mg/animal were orally and intraperitoneally administered to mice and showed immunopotentiating activity stimulating phagocytosis [82]. Methanol extract and polysaccharide fractions from *G. verrucosa* were also antioxidant [82,83]. Aqueous extract from *G. textorii* at a dose of 100 μg/mL did not inhibit platelet aggregation induced by adenosine diphosphate, arachidonic acid or collagen [81]. *G. verrucosa, G. asiatica, G. lichenoides* and others species contain PGE_2_ [47,85], that have physiological effects including hyperthermia, hypotension, smooth muscle dilatation, hyperalgesia and gastric secretion inhibition [114,115] (Table 1).

### 2.5. Gastrointestinal Effects

**Figure 3:**
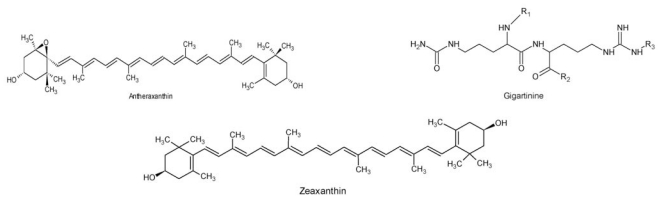
Structure of the compounds found in *G. verrucosa* and *G. chorda.*

Aqueous extract from dried *G. verrucosa* algae or fresh *G. chorda* algae at a dose of 0.5 mg/animal controlled gastrointestinal disorders in mice [48] (Table 1), resulting from zeaxanthin and antheraxanthin [116], carotenoids, pyrimidine 2-amino-4-carboxy, non-alkaloid nitrogen heterocycle [90], steroids, 5-alpha-poriferastane, 3-beta-6-alpha-diol poriferastane, 5-alpha-3-beta-6-beta-diol [51] and gigatinine [85] (Figure 3).

### 2.6. Cardiovascular Effects

90% Ethanol extracts from *G. corticata*, *G. edulis* and *G. verrucosa* showed no cardiovascular effects in dogs (50 mg/kg) [75]. 90 % ethanol extract from *G. edulis* showed diuretic activity [75]. Aqueous extract from *G. lichenoides* was administered intravenously in rats and it was antihypertensive [84]. Tyrosinase inhibition was not induced by methanol extract from *G. arcuata* [86] and aqueous extract from *G. textorii*, 10 μg/mL, was negligable on aldose reductase [83] (Table 1).

### 2.7. Antibiotic Activity

**Figure 4:**
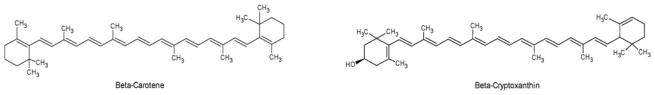
Chemical structure of the steroids isolated from *G. edulis*.

Extracts or ingredients from various algae have shown antibacterial activity *in vitro* against gram-positive and gram-negative bacteria [117]. The agar disc diffusion method for antibacterial susceptibility was used for evaluation and 6 mm discs were impregnated with 20 μL of the extracts and placed in inoculated Muller Hinton agar. Antibacterial activity from chloroform extract of *G. edulis* (Gmelin) Silva was tested against bacterial strains of *Vibrio cholera*, *Staphylococcus aureus, Shigella dysenteriae, Shigella bodii, Salmonella paratyphi, Pseudomonas aeruginosa* and *Klebsiella pneumonia* (Table 1). We observed higher activity for *G. edulis* extract than *S. aureus* extract [12]. Yet it was inactive for *Sporotrichum schenckii, Candida albicans* and *Cryptococcus neoformans* [75]. In the present investigation, the chemical compounds isolated from the species were steroids (carotenoids, β-cryptoxanthin and β-carotene) [118] and carbohydrates [84,85,119] (Figure 4).

Mahasneh *et al.* (1995) demonstrated activity of organic extracts from algae against multi-resistant bacteria to antibiotics [120]. Ethanol extract from *G. debilis* showed antibacterial activity against *S. aureus* but was inactive against *Mycobacterium smegmatis* [92].

95% ethanol extract from whole dried *G. cervicornis* algae was active against *S. aureus* at a concentration of 5.0 mg/mL [89]. Methanol extract from fresh *G. corticata* was active against *Bacillus subtilis*, *Bacillus megaterium, S. aureus* and *Streptococcus viridians* [91].

**Figure 5:**
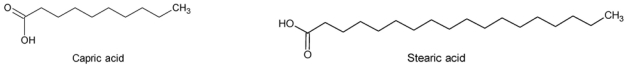
Structure of compounds found in species of *Gracilaria* with antifungal activity.

*G. corticata* and *G. pygmea* did not inhibit the growth of *Aspergillus niger, Fusarium solani, Alternaria solani,* or *Penicillium funiculosum* [91]. Petroleum ether, chloroform and methanol extracts from this seaweed at a concentration of 1.0 μg/units proved to be inactive on the inhibition of penicillinase enzyme [87]. From this specie, stearic lipids and capric acids were isolated [121] (Figure 5).

**Figure 6:**
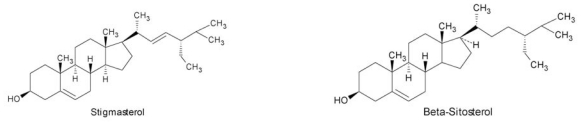

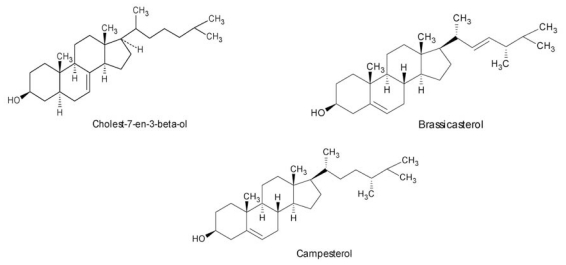
Structure of the steroids isolated from *G. domingensis*.

Ethanol extracts from *G. domigensis* and *G. sjoestedii* showed antibacterial activity against *E. coli* and *S. aureus*. Ethanol extracts from *G. debilis, G. domingensis* and *G. sjoestedii* were active against *Candida albicans* shown by agar plate method [92]; Chloroform, ether and methanol extracts from *G. tikvahiae* were inactive [93]. The growth of *Neurospora crassa* was not inhibited by extracts from *G. sjoestedii* and *G. debilisi*; ethanol extract from *G. domigensis* was active against *Mycobacterium smegmatis* and *Neurospora crassa* [92]. *G. domigensis* has as chemical constituents, polysaccharide CT-1 [122], palmitic acid and steroids (stigmasterol, sitosterol, campesterol, cholest-7-en-3-β-ol and brassicasterol) [52] (Figure 6).

Some studies highlighting antiparasitic activity of seaweeds also were verified. 90 % ethanol extract from *G. corticata* and *G. edulis* were tested against *Entamoeba histolytica* and *Plasmodium berghei* and were not active [75].

### 2.8. Antivirial Activity

**Figure 7:**
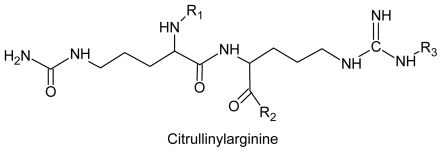
Structure of a compound found in *Gracilaria* sp and *G. bursa-pastoris*.

Extracts from *G. bursa-pastoris* and *Gracilaria* sp were inactive against the *Herpes simplex* 1 virus (HSV) and the human immunodeficiency virus (HIV) when evaluated in cell cultures [96]. Granin BP and citrullinyl-arginine proteins were isolated from these extracts [123,124]. Methanol extract from dried *G. pacifica* at a concentration of 200.0 μg/mL was active against *Sindbis* virus, but was not effective against *H. simplex* 1 when tested at a concentration of 400 μg/mL. Extracts and compounds obtained from *Gracilaria* sp with anti-HIV activity are also active against other retroviruses such as HSV. However, the pharmacodynamic mechanisms of the antiretroviral activity are still unknown because bioactive compounds from seaweed poorly investigated [9] (Table 1) (Figure 7).

## 3. Material and Methods

In this article, some reports about bioactivity of *Gracilaria* algae were reviewed in the specialized literature published up to January 2011. The search was carried out using data banks such as; Biological Abstracts, AlgaeBase, SciFinder Scholar, Pubmed and NAPRALERT (acronym for Natural Products ALERT-University of Illinois in Chicago, USA).

## 4. Conclusions

Algae are abundant in the oceans and represent a rich source of as yet unknown secondary metabolites. In this review, we found only a few studies with complete chemical profiles and pharmacological potential of the *Gracilaria* species. Most studies raised concerns about antimicrobial activity against *Staphylococcus, Streptococcus, Candida* and *Herpes* genus. Others referenced the cytotoxicity bioassays in which these algae species were not active, but they produce various types of prostaglandins and others substances that can be toxic to humans such as gastrointestinal disorders and lethality caused by *G. verrucosa* and *G. edulis*, respectively. To research new drugs it is necessary to evaluate other bioassay models to preserve the safety, efficacy and quality of the end products. In Brazil, there is a great need for toxicological, pharmacological, preclinical and clinical studies, as recommended by the RDC 48/2004.

Finally, we conclude that algae of the *Gracilaria* genus are a potential source for synthesis of new natural medicines. It is important to taxonomically classify and standardize extractions, while identifying the active compounds to attenuate possible environmental interference that could undermine the pharmacochemical profile, and thus generate different pharmacologic effects. In addition, it is important to sensitize corporate researchers and financial agencies to support this cause.

## Figures and Tables

**Table 1:** Bioactivities of marine algae of the *Gracilaria* genus.

Botanical Name	Part used	Type of extract	Bioassays models, organism, dose or route of administration	Result
**Studies of toxicity**				
*Gracilaria bursa-pastoris* (S.G.Gmelin) P.C.Silva	FzDTh	H_2_O Ext.	Cytotoxic activity-cell culture-10.0 μg/mL	Inactive [72]
	FTh	95% EtOH Ext. or CHCl_3_ Ext.	Cytotoxic activity-cell culture-10.0 μg/mL	Inactive [72]
*Gracilaria chorda* (Holmes)	FsO	H_2_O Ext.	Toxicity assessment-mouse-1.2 mg/animal-i.p.	Active [48]
*Gracilaria coronopifolia* (J. Agardh)	FTh	Plant	Toxic effect-human adult-oral	Active [65]
*Gracilaria corticata* (J.Agardh) J.Agardh	Th	50% EtOH-H_2_O Ext.	Toxicity assessment-mouse-DL50 1000 mg/kg-ip	Active [73]
*Gracilaria domingensis* (Kützing) Sonder ex Dickie	DO	90% EtOH Ext.	Cytotoxity-*Artemia salina* L.-200 μg/mL	Active [74]
*Gracilaria edulis* (S.G.Gmelin) P.C.Silva	DTh	Plant	Toxicity effect (death)-human adult-oral	Active [49]
	SDTh	90% EtOH Ext.	Toxicity assessment-mouse-DL50 0.825 mg/kg-i.p.	Active [75]
*Gracilaria foliifera* (Forsskål) Borgesen	DEP	(1:1) EtOH-H_2_O Ext.	Cytotoxic activity-cell culture-dose: dry weight of plant	Active [76]
*Gracilaria textorii* (Suringar) De Toni	FzDO	MeOH Ext.	Cytotoxic activity-cell culture (CA 9 KB)	Inactive [77]
	FsTh	Hexane Ext.	Cytotoxic activity-culture cell (LEUK P 388)-ED 50 > 100 μg/mL	Equivocal [78]
		CCl_4_ Ext.	Cytotoxic activity-culture cell (LEUK P 388)-ED 50 22.2 μg/mL	Equivocal [78]
		CHCl_3_ Ext.	Cytotoxic activity-culture cell (LEUK P 388)-ED 50 32.2 μg/mL	Inactive [78]
*Gracilaria verrucosa* (Hudson) Papenfuss	DO	H_2_O Ext.	Toxicity assessment-mouse-1.2 mg/animal-i.p.	Active [48]
	FzDO	MeOH Ext.	Cytotoxic activity-cell culture (CA 9 KB)	Inactive [77]
	FO	30% EtOH Ext.	Cytotoxic activity-cell culture (CA 9 KB)-10.0 μg/mL	Inactive [79]
		(1:1) CHCl_3_-MeOH Ext.	Cytotoxic activity-cell culture (CA 9 KB)-1.0 μg/mL	Equivocal [79]
	FTh	H_2_O Ext. and 95% EtOH Ext.	Cytotoxic activity-cell culture (LEUK P 388-P 3)-10.0 μg/μL	Inactive [72]

**Effects on the nervous system**				
*Gracilaria corticata* J.Agardh	SDTh	90% EtOH Ext.	Autonomic effects-dog-50 mg/kg-iv	Inactive [75]
			CNS effects-mouse	Inactive [75]
			Analgesic activity-mouse	Inactive [75]
			Anticonvulsant activity-mouse	Inactive [75]
*Gracilaria edulis* (S.G.Gmelin) P.C.Silva	SDTh	90% EtOH Ext.	Autonomic effects-dog-50 mg/kg-iv	Inactive [75]
			CNS effects-mouse	Inactive [75]
			Analgesic activity-mouse	Inactive [75]
			Anticonvulsant activity-mouse	Inactive [75]
*Gracilaria verrucosa* (Hudson) Papenfuss	SDTh	90% EtOH Ext.	CNS effects-mouse	Inactive [75]

**Contraception activity**				
*Gracilaria corticata* J.Agardh	DTh	(1:1) MeOH-CH_2_Cl_2_ Ext.	Embryotoxic effect-pregnant rat-1.0 mg/kg-intragastric	Inactive [80]
	SDTh	90% EtOH Ext.	Antiimplantation effect-pregnant rat-100.0 mg/kg	Inactive [75]
			Spermicidal effect-rat-2.0 %	Inactive [75]
*Gracilaria edulis* (S.G.Gmelin) P.C.Silva	SDTh	90% EtOH Ext.	Antiimplantation effect-pregnant rat-100.0 mg/kg	Inactive [75]
			Spermicidal effect-rat-2.0%	Inactive [75]
*Gracilaria verrucosa* (Hudson) Papenfuss	SDTh	90% EtOH Ext.	Spermicidal effect-rat-2.0%	Inactive [75]

**Anti-inflammatory activity**				
*Gracilaria textorii* (Suringar) De Toni	EP	H_2_O Ext.	Platelet aggregation inhibition (adenosine diphosphate; arachidonic acid or collagen stimulation)-100.0 μg/mL	Inactive [81]
			Venotonic activity (platelet aggregating factor stimulation)-100.0 μg/mL	Inactive [81]
*Gracilaria verrucosa* (Hudson) Papenfuss	DTh	Polysaccharide fraction	Immunostimulant activity-mouse-4.0 mg/animal-i.p.	Active [82]
			Phagocytosis stimulation-mouse-4.0 mg/animal-i.p.	Active [82]
	SDTh	90% EtOH Ext.	Antiinflammatory activity-rat-intragastric	Inactive [75]

**Antioxidant activity**				
*Gracilaria verrucosa* (Hudson) Papenfuss	Plant	MeOH Ext.	Radical scavenging effect (DPPH radicals)-IC50 480.0 μg	Active [83]
	DTh	Polysaccharide fraction	Oxygen radical formation induction-mouse-4.0 mg/animal-i.p.	Active [82]

**Gastrointestinal effects**				
*Gracilaria chorda* (Holmes)	FsO	H_2_O Ext.	Mouse-0.5 mg/animal-gastric intubation and dose 0.5 mg/loop-i.p.	Active [48]
*Gracilaria verrucosa* (Hudson) Papenfuss	DO	H_2_O Ext.	Mouse-0.5 mg/animal-gastric intubation	Active [48]

**Cardiovascular effects**				
*Gracilaria corticata* (J.Agardh) J.Agardh	SDTh	90% EtOH Ext.	Cardiovascular effects-dog-50 mg/kg-iv	Inactive [75]
*Gracilaria edulis* (S.G.Gmelin) P.C.Silva	SDTh	90% EtOH Ext.	Cardiovascular effects-dog-50 mg/kg-iv	Inactive [75]
			Diuretic activity-rat-intragastric	Active [75]
*Gracilaria lichenoides* (Greville)	EP	H_2_O Ext.	Antihypertensive activity-rat-iv	Active [84]
	FsTh	H_2_O Ext.	Antihypertensive activity-rat-iv	Active [85]
*Gracilaria verrucosa* (Hudson) Papenfuss	SDTh	90%EtOH Ext.	Cardiovascular effects-dog-50 mg/kg-iv	Inactive [75]

**Hypoglycemic activity**				
*Gracilaria corticata* J.Agardh	SDTh	90% EtOH Ext.	Rat-250 mg/kg – intragastric	Inactive [75]
*Gracilaria edulis* (S.G.Gmelin) P.C.Silva	SDTh	90% EtOH Ext.	Rat-250.0 mg/kg – intragastric	Inactive [75]

**Anti-enzymes activity**				
*Gracilaria arcuata* (Zanardini)	DTh	MeOH Ext.	Tyrosinase inhibition-0.33 mg/mL	Inactive [86]
*Gracilaria corticata* (J.Agardh) J.Agardh	DO	PET Ether Ext.; CHCl_3_ Ext. or MeOH Ext.	Penicillinase inhibition-1.0 μg/units	Inactive [87]
*Gracilaria textorii* (Suringar) De Toni	EP	H_2_O Ext.	Aldose reductase inhibition-10.0 μg/mL	Inactive [81]
	FzDO	MeOH Ext.	Cyclic AMP phosphodiesterase inhibition	Inactive [77]
*Gracilaria verrucosa* (Hudson) Papenfuss	FzDO	EtOAc Ext.	Lipase inhibition	Equivocal [88]
		MeOH Ext.	Cyclic AMP phosphodiesterase inhibition	Inactive [77]

**Respiratory effects**				
*Gracilaria corticata* (J.Agardh) J.Agardh	SDTh	90% EtOH Ext.	Respiratory depressant-dog-50 mg/kg-iv	Inactive [75]
*Gracilaria edulis* (S.G.Gmelin) P.C.Silva	SDTh	90% EtOH Ext.	Respiratory depressant-dog-50 mg/kg-iv	Inactive [75]
*Gracilaria verrucosa* (Hudson) Papenfuss	SDTh	90% EtOH Ext.	Respiratory depressant-dog-50.0 mg/kg-iv	Inactive [75]

**Spasmolytic activity**				
*Gracilaria corticata* (J.Agardh) J.Agardh	SDTh	90% EtOH Ext.	Spasmolytic activity-guinea pig	Inactive [75]
*Gracilaria edulis* (S.G.Gmelin) P.C.Silva	SDTh	90% EtOH Ext.	Negative chronotropic effect-dog-50.0 mg/kg-iv	Inactive [75]

**Antibacterial activity**				
*Gracilaria cervicornis* (Turner) J.Agardh	DEP	95% EtOH Ext.	Agar plate-*Staphylococcus aureus*-5.0 mg/mL	Active [89]
			Agar plate-*Proteus vulgaris; Escherichia coli; Aspergillus fumigates; Candida albicans; Pseudomonas aeruginosa; Streptococcus pyogenes*- 50.0 mg/mL	Inactive [89]
*Gracilaria corticata* (J.Agardh) J.Agardh	DO	PET Ether Ext.; CHCl_3_ Ext. or MeOH Ext.	Agar plate-*Staphylococcus aureus; Escherichia coli*-MIC >200 μg/mL	Inactive [90]
	FsO	MeOH Ext.	Agar plate-*Escherichia coli; Salmonella paratyphi A; Salmonella paratyphi B; Shigella sonnei*	Inactive [91]
			Agar plate-*Bacillus subtilis; Staphylococcus aureus; Bacillus megaterium; Streptococcus viridans*	Active [91]
	SDTh	90% EtOH Ext.	Agar plate-*Klebsiella pneumonia; Pseudomonas aeruginosa; Staphylococcus aureus; Escherichia coli; Streptococcus faecalis*	Inactive [75]
*Gracilaria debilis* (Forsskål) Borgesen	DO	95% EtOH Ext.	Agar plate-*Escherichia coli; Staphylococcus aureus*	Active [92]
			Agar plate-Mycobacterium smegmatis Inactive	[92]
*Gracilaria domingensis* (Kützing) Sonder ex Dickie	DO	95% EtOH Ext.	Agar plate-*Escherichia coli; Staphylococcus aureus*	Active [92]
		Acetone Ext. or Ether Ext.	Agar plate-*Escherichia coli; Staphylococcus aureus*	Inactive [92]
		95% EtOH Ext. or Acetone Ext.	Agar plate-*Mycobacterium smegmatis*	Active [92]
*Gracilaria edulis* (S.G.Gmelin) P.C.Silva	SDTh	90% EtOH Ext.	Agar plate-*Escherichia coli; Streptococcus faecalis; Staphylococcus aureus; Pseudomonas aeruginosa; Klebsiella pneumoniae*	Inactive [75]
*Gracilaria pygmea* (Borgesen)	FsO	MeOH Ext.	Agar plate-*Bacillus subtilis; Staphylococcus aureus; Escherichia coli; Salmonella paratyphi A; Streptococcus viridans; Shigella sonnei; Salmonella paratyphi B*	Inactive [91]
			Agar plate-*Bacillus megaterium*	Active [91]
*Gracilaria sjoestedii* (Kylin)	DO	95% EtOH Ext.	Agar plate-*Escherichia coli; Staphylococcus aureus*	Active [92]
			Agar plate-Mycobacterium smegmatis	Inactive [92]
*Gracilaria tikvahiae* McLachlan	DEP	CHCl_3_ Ext. or EtOH Ext.	Agar plate -*Staphylococcus aureus*	Active [93]
			Agar plate-*Streptococcus faecalis; Pseudomonas aeruginosa*	Inactive [93]
*Gracilaria verrucosa* (Hudson) Papenfuss	FTh	**	Agar plate-*Vibrio marinofulvis; Micrococcus imfimus; Pseudomonas atlantica*-40.0 μg/μL	Inactive [94]
	Th	70% EtOH Ext.	Antiphage activity-agar plate-Bacteriophage T 1; Bacteriophage T 2; Bacteriophage T 4; Bacteriophage T 7; Bacteriophage MS 2; Bacteriophage PHI-CHI 174-0.50 μg/mL	Inactive [95]

**Antifungal activity**				
*Gracilaria corticata* (J.Agardh) J.Agardh	FsO	MeOH Ext.	Agar plate-*Aspergillus niger; Fusarium solani; Alternaria solani; Penicillium funiculosum*	Inactive [91]
	SDTh	90% EtOH Ext.	Agar plate-*Sporotrichum schenckii; Cryptococcun neoformans; Candida albicans; Trichophyton mentagrophytes; Aspergillus fumigates*	Inactive [75]
*Gracilaria debilis* (Forsskål) Borgesen	DO	95% EtOH Ext.	Agar plate-*Candida albicans*	Active [92]
			Agar plate-*Neurospora crassa*	Inactive [92]
*Gracilaria domingensis* (Kützing) Sonder ex Dickie	DO	95% EtOH Ext. and Acetone Ext.	Agar plate-*Candida albicans; Neurospora crassa*	Active [92]
		Ether Ext.	Agar plate-*Candida albicans*	Inactive [92]
*Gracilaria edulis* (S.G.Gmelin) P.C.Silva	SDTh	90% EtOH Ext.	Agar plate-*Sporotrichum schenckii; Candida albicans; Cryptococcus neoformans; Trichophyton mentagrophytes; Aspergillus fumigates*	Inactive [75]
*Gracilaria pygmea* (Borgesen)	FsO	MeOH Ext.	Agar plate-*Aspergillus niger; Fusarium solani; Alternaria solani; Penicillium funiculosum*	Inactive [91]
*Gracilaria sjoestedii* (Kylin)	DO	95% EtOH Ext.	Agar plate-*Candida albicans*	Active [92]
			Agar plate-*Neurospora crassa*	Inactive [92]
*Gracilaria tikvahiae* McLachlan	DEP	CHCl_3_ Ext. and EtOH Ext.	Agar plate-*Candida albicans*	Active [93]

**Antiviral activity**				
*Gracilaria bursa-pastoris* (S.G.Gmelin) P.C.Silva	FzDTh	**	Cell culture-*Herpes simplex* 1 and HIV Virus	Inactive [96]
*Gracilaria corticata* (J.Agardh) J.Agardh	Th	50% EtOH-H_2_O Ext.	Cell culture-*Semlicki-forest* Virus-0.05 mg/mL	Equivocal [73]
			Cell culture-*Ranikhet* and *Vaccinia* Virus-0.05 mg/mL	Inactive [73]
	SDTh	90% EtOH Ext.	Cell culture-*Ranikhet* Virus	Inactive [75]
*Gracilaria edulis* (S.G.Gmelin) P.C.Silva	SDTh	90% EtOH Ext.	Cell culture-*Semlicki-forest* and *Ranikhet* Virus	Inactive [75]
*Gracilaria pacifica* (I. A. Abbott)	DO	MeOH Ext.	Cell culture-*Herpes simplex* 1 Virus-400.0 μg/mL	Inactive [97]
			Cell culture-*Virus sindbis*-200.0 μg/mL	Active [97]
*Gracilaria* species	FzDTh	**	Cell culture-Herpes simplex 1 and HIV Virus	Inactive [96]
*Gracilaria textorii* (Suringar) De Toni	FzDO	MeOH Ext.	Cell culture-*Herpes simplex* 1 Virus	Inactive [77]
	Th	H_2_O Ext.	Cell culture-HIV Virus-MIC > 1000 μg/mL	Inactive [98]
	Fresh	MeOH Ext.	Epstein-Barr virus early antigen activation inhibition (telocidin b-4 induced	** [99]
	Th		Epstein-Barr virus induced activation)-4.0 μg/mL	
*Gracilaria verrucosa* (Hudson) Papenfuss	FzDO	MeOH Ext.	Cell culture-*Herpes simplex* 1 Virus	Inactive [77]

**Antiprotozoal activity**				
*Gracilaria corticata* (J.Agardh) J.Agardh	SDTh	90% EtOH Ext.	Agar plate-*Entamoeba histolytica; Plasmodium berghei*	Inactive [75]
*Gracilaria edulis* (S.G.Gmelin) P.C.Silva	SDTh	90% EtOH Ext.	Agar plate-*Entamoeba histolytica; Plasmodium berghei*	Inactive [75]

**Allelophatic activity**				
*Gracilaria compressa* (C.Agardh) Greville	DEP	95% EtOH Ext.	Agar plate-*Helianthus tuberosus*-dose: dry weight of plant	Active [76]
*Gracilaria foliifera* (Forsskål) Borgesen	DEP	H_2_O Ext.	Agar plate-*Helianthus tuberosus*-dose: dry weight of plant	Active [76]
*Gracilaria verrucosa* (Hudson) Papenfuss	DEP	95% EtOH Ext.	Agar plate-*Helianthus tuberosus*-dose: dry weight of plant	Active [76]

Legend: DEP = Dried entire plant; DO = Dried organism; DTh = Dried thallus; EP = Entire plant; FO = Frozen organism; FsO = Fresh organism; FsTh = Fresh thallus; FTh = Frozen thallus; FzDO = Freeze dried organism; FzDTh = Freeze Dried thallus; SDTh = Shade dried thallus; Th = Thallus; PET Ether);

** = Not stated.
